# Biocontrol properties from phyllospheric bacteria isolated from *Solanum lycopersicum* and *Lactuca sativa* and genome mining of antimicrobial gene clusters

**DOI:** 10.1186/s12864-022-08392-0

**Published:** 2022-02-21

**Authors:** Claudia Y. Muñoz, Lu Zhou, Yunhai Yi, Oscar P. Kuipers

**Affiliations:** Department of Molecular Genetics, University of Groningen, Groningen, The Netherlands

**Keywords:** Biocontrol, Phyllosphere, Bacillus, Paenibacillus, Antimicrobials, biosynthetic gene clusters, NRPs, bacteriocins, RiPPs

## Abstract

**Background:**

Biocontrol agents are sustainable eco-friendly alternatives for chemical pesticides that cause adverse effects in the environment and toxicity in animals including humans. An improved understanding of the phyllosphere microbiology is of vital importance for biocontrol development. Most studies have been directed towards beneficial plant-microbe interactions and ignore the pathogens that might affect humans when consuming vegetables. In this study we extended this perspective and investigated potential biocontrol strains isolated from tomato and lettuce phyllosphere that can promote plant growth and potentially antagonize human pathogens as well as plant pathogens. Subsequently, we mined into their genomes for discovery of antimicrobial biosynthetic gene clusters (BGCs), that will be further characterized.

**Results:**

The antimicrobial activity of 69 newly isolated strains from a healthy tomato and lettuce phyllosphere against several plant and human pathogens was screened. Three strains with the highest antimicrobial activity were selected and characterized (*Bacillus subtilis* STRP31, *Bacillus velezensis* SPL51, and *Paenibacillu*s *sp.* PL91). All three strains showed a plant growth promotion effect on tomato and lettuce. In addition, genome mining of the selected isolates showed the presence of a large variety of biosynthetic gene clusters. A total of 35 BGCs were identified, of which several are already known, but also some putative novel ones were identified. Further analysis revealed that among the novel BGCs, one previously unidentified NRPS and two bacteriocins are encoded, the gene clusters of which were analyzed in more depth.

**Conclusions:**

Three recently isolated strains of the Bacillus genus were identified that have high antagonistic activity against lettuce and tomato plant pathogens. Known and unknown antimicrobial BGCs were identified in these antagonistic bacterial isolates, indicating their potential to be used as biocontrol agents. Our study serves as a strong incentive for subsequent purification and characterization of novel antimicrobial compounds that are important for biocontrol.

**Supplementary Information:**

The online version contains supplementary material available at 10.1186/s12864-022-08392-0.

## Background

There is an increasing demand by consumers for nutritious foods that improve physical performance and reduce risks of disease. Vegetables represent a widely consumed food worldwide. *Solanum lycopersicum* better known as tomato is one of the most important vegetable plants in the world [1]. Tomato is a healthy food that supplies a wide range of vitamins needed for the organism, since it contains high levels of zinc, potassium, anthocyanins and lycopene, which provide a high antioxidant power. Along with other compounds they reduce risk of contracting cancer, among other benefits according to several epidemiological-food- and health studies [1, 2]. On the other hand, *Lactuca sativa* better known as lettuce is another important crop with a growing interest from people due to its healthy and beneficial properties and richness in antioxidants (e.g., vitamins C, E and carotenoids) [3].

Agriculture in the last century has faced multiple challenges, including the need to produce more food to feed a growing population, adapting to climate change, controlling crop diseases that cause significant losses, and adopting more efficient and sustainable production methods. An increased number of fungi, bacteria and viruses are causing plant diseases, several of which are the reason of major economic losses [4]. For this reason, food security has become one of the main points of attention in human-driven development, and therefore any plant pathogen causing substantial crop yield losses needs to be minimized [5]. Currently, tomato and lettuce production losses due to biotic agents (insect pests, microbe -or- virus caused diseases and weeds) have been controlled mainly by spraying crops with a vast amount of synthetic chemical pesticides. However, the extensive use of them caused adverse side effects which represent a serious threat to living organisms and the environment. In addition, for many plant pathogens, fungicide-resistant populations have made many fungicides ineffective [6].

Some human pathogens, such as *Bacillus cereus*, attach and form biofilms on lettuce leaf surfaces posing a risk of causing disease in humans upon consumption [7]. *Klebsiella pneumoniae* and *Escherichia coli* are other known human pathogens that have been reported as causative agents of foodborne diseases, being found in different sources including raw vegetables [8]. Contamination by pathogenic bacteria of vegetables can occur during the harvesting period, post harvesting, handling, storage, transportation, and processing by customers. To ensure safety level of vegetables and others, research of biocontrol agents against human pathogens is needed as well.

It is now well established that plant-associated microorganisms play essential roles in plant health and development and contribute to the environmental equilibrium [9]. The use of beneficial microorganisms is a promising method to fight against crop diseases and increase yields to ensure sufficient crop production [10]. The structural and functional analysis of microbial genomes and the proteins encoded by genes of important plant-associated microbes, which can be possible biocontrol agents will provide insights into several aspects of these molecular interactions and will be crucial for the development of more efficient control measures [11].

Historically, research investigating the factors associated with plant microbe interactions has focused on the rhizosphere, which comprises the area in the soil around plant roots, but much less is known about the phyllosphere. The phyllosphere represents the aboveground or aerial parts of the plants dominated by the leaves in contrast to the rhizosphere. Environmental factors, including UV radiation, changes in relative humidity, temperature, leaf wetness, pollution, nitrogen fertilization as well as biotic factors, such as leaf age and the presence of other microorganisms are factors that microbes endure in such environments [12].

Gram-positive, aerobic spore forming bacteria, like *Bacillus* and *Paenibacillus spp.*, have been widely reported to be effective in stimulating plant growth and are well known as producers of a broad array of antimicrobials having between 5 and 8% of the total genome devoted to the biosynthesis of secondary metabolites [13].

The antimicrobials production of *Bacillus* and closely related species is highly diverse, depending on their biosynthesis pathways, and chemical nature. Antimicrobial compounds can be classified into bacteriocins (both linear and ribosomally synthesized and postranslationally modified peptides (RiPPs)), non- ribosomally synthesized peptides (NRPs), and Polyketides [14]. NRPs and PKs natural products are synthesized via multi domain mega enzymes known as non-ribosomally synthesized peptides synthases (NRPSs) and polyketide synthases (PKSs) which are arranged into units called ‘modules’ that work in an assembly-line system to build polymeric peptide chains with a determined function [15]. PKSs gather small acetic acid-type acyl construction blocks into polyketides through C–C bonds, and NRPSs gather amino acids into peptides through amide bonds. NRPSs and PKSs employ a similar strategy for the biosynthesis of different classes of natural products [16]. On the other hand, bacteriocins are ribosomally synthesized. Bacteriocin BGCs are smaller than those of the previous mentioned compounds, and carry one or more precursor peptide gene(s), which allows for a finer prediction of the end product structure based on the properties of enzymes involved in their biosynthesis and on the chemical structure of the initial peptide substrate. Bacteriocins can be classified in peptides that undergo post-translational modifications (class I) also known as Ribosomally synthesized and Post-translationally modified Peptides (RiPPs), or largely unmodified peptides, that sometimes contain disulfide bonds (class II) [17].

Reported and well-known NRPs antimicrobials produced by *B. subtilis* and *B. velezensis* are cyclic lipopeptides exhibiting strong surfactant and antimicrobial activities, such as surfactins, bacillibactins and fengycins [14, 18]. *B. velezensis*, the biocontrol model also employs polyketides such as macrolactin, bacillaene, and difficidin, which play significant roles in both pathogen suppression and plant growth promotion [19]. NRPs like fusaricidin and polymyxin produced by *Paenibacillus* strains, contributes to antagonism against phyopathogens like *Erwinia spp.* [20], while well studied *Bacillus*-originated bacteriocins include subtilosin and subtilomycin [21].

In the last decade volatile compounds (VOCs) produced by some plant-associated bacteria and their biological function, have attracted increased attention. Among other characteristics, they have been proved to have the potential as antimicrobials and have plant growth promoting properties. The VOCs 2,3-butanediol and acetoin, could trigger growth promotion in *Arabidopsis thaliana* rhizosphere. *Bacillus megaterium* XTBG34 produces 2-pentylfuran which promotes the growth of *Arabidopsis thaliana* plants after 15 days of treatment [22, 23]. In our study we aimed to isolate and screen novel phyllospheric bacteria with antimicrobial properties, and further mined into their genomes to identify known or novel biosynthetic gene clusters (BGCs) that are potentially involved in phytopathogen, and plant-originated human pathogen antagonism.

## Results

### Isolation of phyllosperic strains and antimicrobial activity against pathogens

A total of 69 strains were isolated from internal and external leaf tissue from tomato and lettuce plants in early growth stage coming from a small farm in Roden, The Netherlands (Table S1). The antimicrobial activity of the 69 strains was first evaluated against five major tomato and lettuce pathogens, i.e., *Rhizoctonia solani* [24], *Botrytis cinerea* [25], and *Phytium ultimum* [26] and two gram-negative bacterial pathogens, i.e. *Pseudomonas syringae pv* tomato DC300 [27], and *Erwinia corotovora* [28, 29], covering the two major classes of pathogens, i.e. fungi and Gram-negative bacteria. The three most outstanding strains with the highest antimicrobial activity were selected and their antimicrobial spectrum was extended, to evaluate activity against a wider range of pathogens and to further evaluate properties of plant growth promotion.

**Fig. 1:**
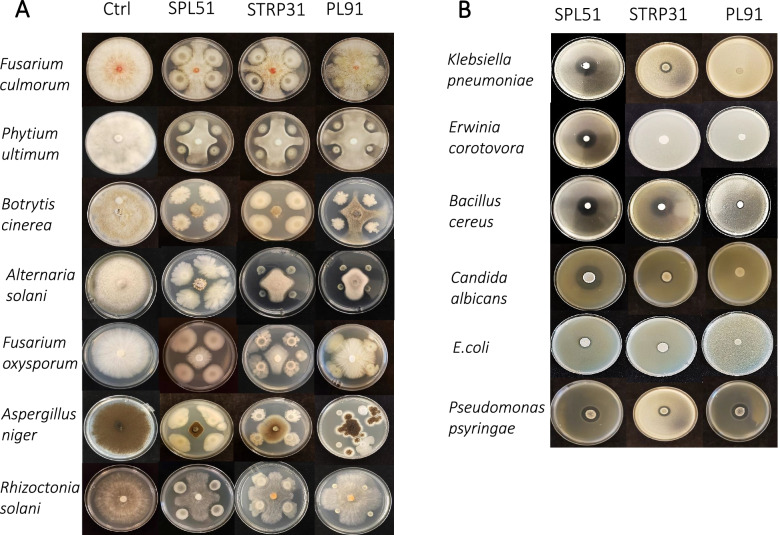
**A** Fungal inhibition assay with selected strains *B. velezensis* SPL51, *B. subtilis* STRP31 and *Paenibacillus sp.* PL91. **B** Bacterial inhibition assay with selected strains *B. velezensis* SPL51, *B. subtilis* STRP31 and *Paenibacillus sp*. PL91

In addition, raw and not-processed vegetables, such as tomato and lettuce in salads, are considered as a source of food-borne diseases as well. Some human pathogens can inhabit plants as secondary hosts. In the present study some human pathogens (*Klebsiella pneumoniae* [30], *Bacillus cereus* [31] *Escherichia coli* [32] and *Candida albicans* [33]) were also included in the antimicrobial activity tests as indicators. Three bacteria with the highest antimicrobial activity were selected: *Bacillus subtilis* STRP31 isolated from tomato, and *Bacillus velezensis* SPL51, and *Paenibacillus sp.* PL91 both isolated from lettuce. These strains can antagonize Gram-positive pathogenic bacteria, Gram-negative pathogenic bacteria, a yeast, and fungal pathogens (Table 1; Fig. 1). The other 66 strains showed a significant lower activity towards bacterial and fungal pathogens tested and had a more limited spectrum of activity.

**Table 1:** Antimicrobial activity of phyllospheric bacterial strains *Bacillus velezensis* SPL51, *Bacillus subtilis* STRP31 and *Paenibacillus sp*. PL91

Pathogen types	Species or strains	SPL51	STRP31	PL91
Fungi	*Rhizoctonia solani* AG2-2 IIIB	●	●	●
	*Botrytis cinerea BO5.10*	●	●	●
	*Verticillium dahlia JR2*	●	○	○
	*Fusarium culmorum* PV	●	●	●
	*Fusarium oxysporum*	●	●	●
	*Alternaria solani*	●	●	●
	*Septoria lycopersici*	●	○	○
	*Aspergilus niger*	●	●	○
Oomycete	*Phytium ultimum P17*	●	●	●
Yeast	*Candida albicans*	++	+	-
Gram-negative bacteria	Escherichia coli WA321 *	+	+	-
	Klebsiella pneumoniae *	++	+	-
	*Pseudomonas syringae pv. tomato DC300*	++	+	++
	*Erwinia corotovora subsp. brasiliensis LMG21371*	++	-	-
Gram-positive bacteria	Bacillus cereus ATCC14579 *	++	+	++

In the antibacterial assay, no inhibition (−), inhibitory zone < 5 mm (+), inhibitory zone ≥5 mm (++). In the antifungal assay, no inhibition (○), inhibition (●). * Human pathogens

### Genome sequencing of the selected strains and phylogenetic analyses

**Fig. 2:**
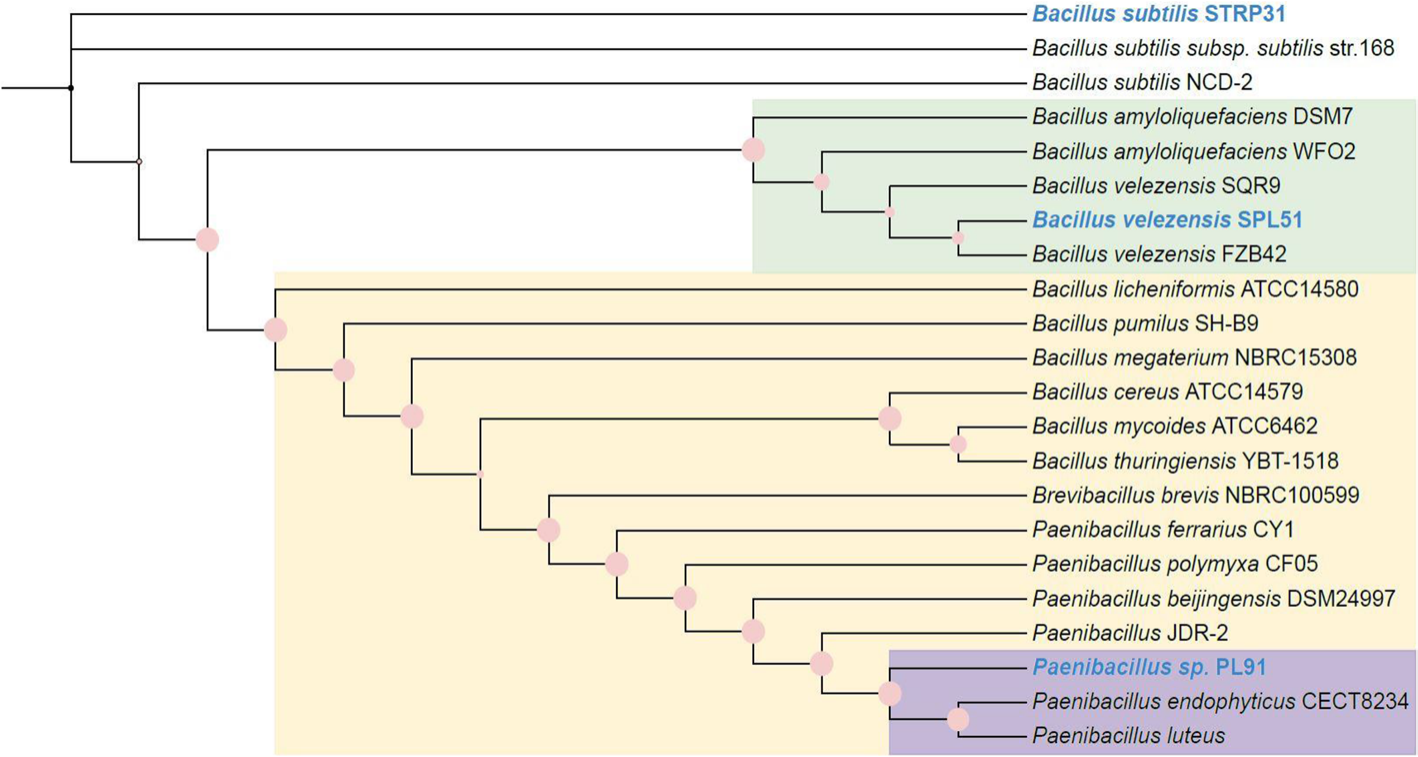
Phylogenetic analysis of the selected isolated strains with antimicrobial activity in this study. The sequences of reference strains were retrieved from the NCBI database (Table S3 for accession number). The reference strains are in black, and our sequenced strains are highlighted with blue. The pink dots on branches represent bootstrapping values, ranging from 84 to 100

The genomes of the selected strains were sequenced, and their DNA sequences were reported previously [34]. Phylogenetic analysis using these whole-genome sequences was conducted with GTDB-Tk v1.5.0 [35] and a phylogenetic dendrogram was constructed with the obtained sequences by IQ-TREE and visualized by iTOL web-based tool [36] (Fig. 2). The species names of the three strains were designated as their most closely related strains namely *B. velezensis* SPL51, *B. subtilis* STRP31, and unidentified *Paenibacillus sp.* PL91.

## Plant growth promotion effect

**Fig. 3:**
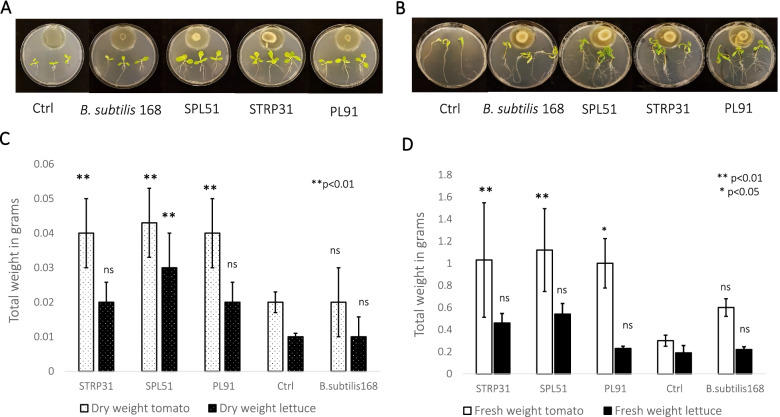
Plant growth promotion by volatiles. **A** Phenotypic changes in lettuce seedlings exposed to volatiles emitted by SPL51, STP31, and PL91 strains inoculated on agar LB medium (10 µl OD=1.0), the control LB agar only, and *B. subtilis* 168 used as second control. Pictures were taken 7 days after exposure. **B** Phenotypic changes in tomato seedlings exposed to volatiles emitted by SPL51, STP31, PL81, and PL91 strains inoculated on agar LB medium (10 µl OD=1.0) the control LB agar only, and *B. subtilis* 168 used as second control. Pictures were taken 10 days after exposure. **C** Biomass dry weight (mean ± standard error [SE], *n* = 4). **D** Biomass, fresh weight (mean ± standard error [SE], *n* = 4). (ns= non-significant, * **= significant, both compared to Ctrl)

In vitro experiments were conducted to investigate the effect of volatile organic compounds (VOCs) produced by the three strains (SPL51, STRP31 and PL91) on plant growth of tomato and lettuce plants. Comparisons were made using *B. sutbilis 168* as negative control. We observed that the bacterial isolates exerted a significant influence on the increase of plant biomass of tomato and lettuce plants. The plant growth promoting potential of VOCs produced by SPL51, STRP31 and PL91 is depicted in Fig. 3.

### Genome mining for BGCs

**Fig. 4:**
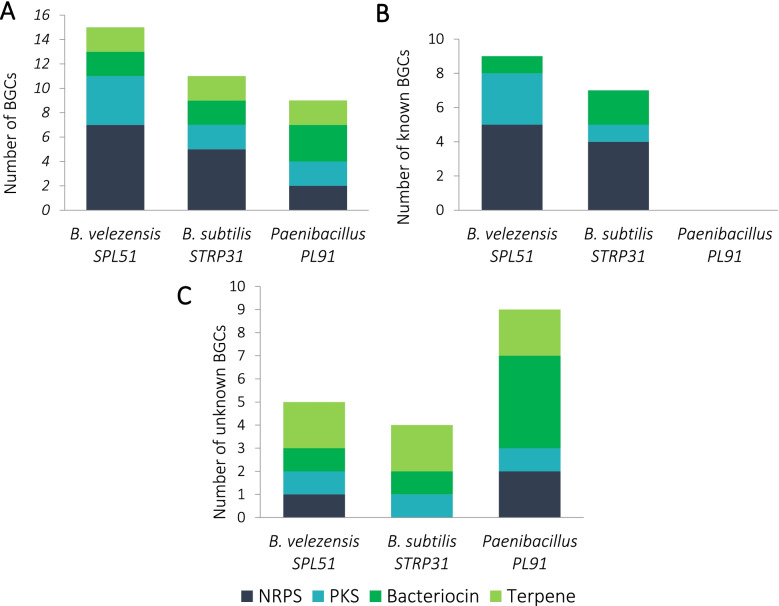
Numbers of BGCs harbored by the strains. **a** total number of BGCs in the strains. **b** number of reported BGCs in the genomes of strains. **c** number of unknown BGCs found in the strains. BGCs that have different numbers of genes or show equal or less than 80% protein identity to the reported ones were regarded as novel

A total of 39 gene clusters were found within the three selected strains (Fig. 4A). Among the three strains NRPSs were most abundant, followed by PKSs. The majority of the BGCs found in *B. subtilis* STRP31 and *B. velezensis* SPL51 are already known (Fig. 4B). The known BGCs encoded in the genomes are: anabaenopeptin [37], bacilysin [38], bacillibactin [39], difficidin [40], fengycin [41], bacillaene [42], macrolactin H [43], plantazolicin [44], surfactin [45], subtilomycin [46], subtilosin A [47], paeninodin [48] (additional file S2). The highest number of the unknown BCGs belong to strain *Paenibacillus sp. PL91 having nine unknown BGCs*, among them are: two NRPSs, one PKs, four bacteriocins and two terpenes. (Fig. 4C).

### Putative novel BGCs

**Fig. 5:**
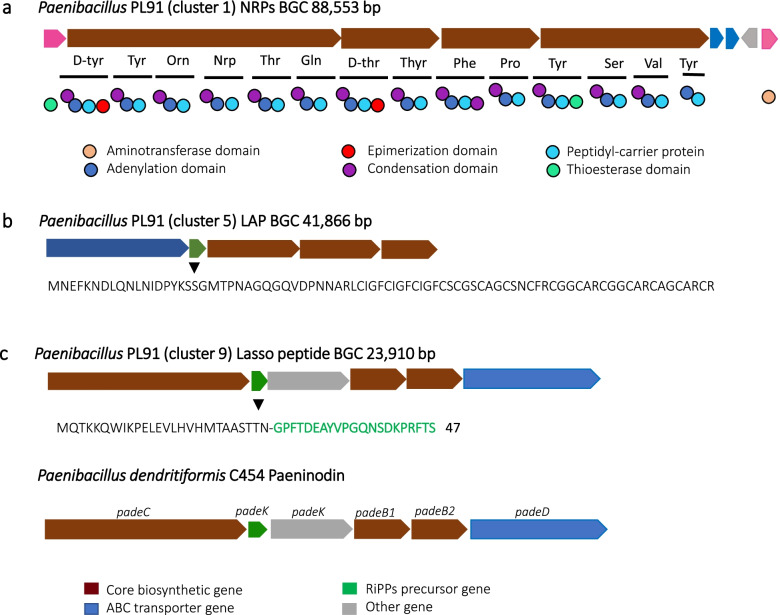
Potential novel BCGs found in the genomes of the selected strains. **a** an NRPS present in *Paenibacillus* PL91. **b** a LAP present in *Paenibacillus* PL91, **c** A lasso peptide harbored by *Paenibacillus* PL91 compared to a Paeninodin lasso peptide produced by *Paenibacillus dendritiformis* C454. The arrows represent the coding regions of the genes identified in the genomes

After genome mining, the three more interesting, potentially novel BGCs were chosen based on sequence similarity to other compounds and after comparing the precursor peptides. Selected BGCs are listed in Fig. 5. In the genome of *Paenibacillus sp.* PL91 a modular NRPS gene cluster with all essential modules (starting module, elongation module, termination module) was found (Fig. 5a). The BGC consists of nine genes and has a total size of 88 kb, it shows 25% of sequence similarity to the cyclic antimicrobial decapeptide tyrocidine, produced by *Brevibacillus brevis* NBRC, which exhibits antifungal and antibacterial activity against Gram-positive bacteria. Tyrocidine A, B and C produced by *Brevibacillus* spp. share structural similarities, summarized as Cyclo (Phe - Pro2 - Phe/Trp3 - Phe/Trp4 - Asn5 - Gln6 - Tyr7 - Val8 - Orn9 - Leu10). Residues 1,2, and 5 to 10 are conserved among the three tyrocidine members produced by *Brevibacillus* [49]. However, in the NRPs BGC found in PL91 neither the order of genes nor the predicted amino acid composition shows similarity to tyrocidine, indicating the likely novelty of the final product.

Another BGC found in *Paenibacillus sp.* PL91 was a linear azol(in)e-containing peptide (LAP), an important subgroup of RiPPs with a distinguishing heterocyclic ring of oxazoles and thiazoles derived from serine/ threonine and cysteine by enzymatic cyclodehydration and dehydrogenation [50]. This BGC consists of five genes and has a total size of 41 kb. The precursor peptide encoded by the core biosynthetic gene has 83 amino acids and shows no similarity with sequences of other bacteriocins. Since it is a bacteriocin it might be possible that it is partially responsible for the antimicrobial activity against Gram-positive bacteria, which needs to be further investigated.

An interesting lasso peptide BGC, discovered in *Paenibacillus* sp. PL91, has 80% sequence similarity to paeninodin. Paeninodin was firstly discovered in *Paenibacillus dendritiformis* C454. It was firstly reported in the year 2016 for being a novel lasso peptide tailored by a new class of kinases [48]. Lasso peptide biosynthesis requires at least three genes, referred to as the A, B, and C proteins. The A gene encodes the precursor peptide, which is modified by the B and C proteins into the mature natural product [51]. The Lasso peptide BGC found in strain PL91 consists of six genes and has a total size of 23 kb. When we compared lasso peptide BGC in PL91 with the paeninodin lasso peptide, we found they have a highly similar gene organization, and the same number genes too. However, the suggested functionality of the genes is not completely the same. After further analysis with Blastp, the results reveal that the first gene might be an isopeptide-bond forming cyclase protein with cyclization function. The second gene corresponds to the precursor gene. The corresponding precursor peptide compared to the paeninodin produced by *Paenibacillus dendritiformis* C454 is not the same, thus, this finding suggests a probable novelty of the final product (Fig. 5c). The third gene is suggested to correspond to an aldolase, which is also present in other *Paenibacillus* spp., while the third gene of *Paenibacillus dendritiformis* is *Pade-K*, a gene encoding a kinase, which is not present in the lasso peptide BGC of *Paenibacillus sp*. PL91. The fourth gene corresponds to be a PqqD family chaperone with a possible function as a maturation enzyme. The fifth gene appears to be a B2 protein, with a proposed function as a maturation enzyme as well, B2 part of ‘split B’, protease. Finally, the sixth gene corresponds to be an ABC transporter, with a proposed export function (Table 2).

**Table 2:** Proposed functions of ORFs in the putative lasso peptide biosynthetic gene cluster identified by genome mining in *Paenibacillus* sp. PL91 and reported *Bacillus Dendrifitormis* C454

Gene	Paeninodin *Paenibacillus dendritiformis C454*	Gene	Lasso peptide BGC *Paenibacillus* PL91
*pade C*	Maduration enzyme, cyclization, adenylation	1	Isopeptide bond cyclase
*padeA*	Precursor peptide	2	Precursor peptide
*padeK*	Putative kinase	3	Aldolase
*padeB1*	Maturation enzyme PqqD superfamily B1 part of “split B”, protease	4	Maturation enzyme PqqD chaperone
*padeB2*	Maturation enzyme, transglutaminase like superfamily B2 part of “split B”, protease	5	B2 protein maturation enzyme
*padeD*	Export ABC type transport system	6	ABC transporter ATP-binding protein

## Discussion

In this study we have identified three promising new biocontrol strains, isolated from tomato and lettuce leaves. In addition to their antagonistic activity against several plant and human pathogens, they exert a plant growth promotion effect in tomato and lettuce by the production of VOCs. In a previous study a genome-based analysis from rhizosphere bacteria revealed the presence of several biosynthetic gene clusters (BGCs) for secondary metabolite production which further helped to the pave the road for antimicrobial discovery [52]. In the present study we choose to study a different region, the phyllosphere, which has historically lagged behind our knowledge of the microbiology of the rhizosphere. Insights into the microbial phyllosphere populations and their antimicrobial potential will help us to have a better understanding of the phyllosphere microbiota and develop applications in the promotion of plant growth and plant protection.

We show the presence of a large variety and number of biosynthetic gene clusters among the three selected strains, which were quite different from the best strains found in the rhizosphere [52]. However, various BGCs identified in *Bacillus velezensis* SPL51 and *Bacillus subtilis* STRP31 were already known (so called rediscovery), and both strains represent two well studied species with high potential as biocontrol agents to be applied in agriculture, although the ones we isolated could have additional advantages. *Paenibacillus sp.* PL91 harbored most of the unknown BGCs, including a potentially novel NRPs, and two bacteriocin encoding BGCs (one LAP and one lasso peptide). We suspect that the novel BGCs found in this study have antibacterial and (or) antifungal activity from the antimicrobial assay realized, but this need to be confirmed by future experiments.

Bacteriocins have many properties including their potency (as determined in vitro and in vivo), their low toxicity, the availability of both broad- and narrow-spectrum peptides, the possibility of in situ production by probiotics and the fact that they can be bioengineered. One of the novel BGCs found is a lasso peptide BGC. Lasso peptides are characterized by having peptide bonded proteinogenic amino acids with an extra linkage between the N- terminus of the peptide and an aspartate or glutamate sidechain, which give them the resembling characteristic topology of a slipknot. Most lasso peptides carry Gly, Cys, Ser or Ala at the N-terminus, and their structure confers them an excellent stability against thermal, chemical, and proteolytic degradation. These features make them attractive for the generation of more potent compounds by engineering. Some lasso peptides are known for their antimicrobial activity, enzyme inhibition, anticancer properties, and antiviral activity. Microcin J25, specialicin, achromosin, sphaericin, actinokineosin, lassomycin are some examples of lasso peptides with antimicrobial activity [53, 54]. This makes them attractive not only for biocontrol purposes but also for pharmaceutical research. The lasso peptide present in strain PL91 has significant differences when compared to other known lasso peptides, suggesting that it is a novel variant of paeninodin, thus making it highly interesting for antimicrobial activity and drug discovery.

On the other hand, the LAP BGC found in PL91 has no similarity with any other known compound. LAPs main components include a precursor peptide (A), and a heterotrimeric enzyme complex composed of a dehydrogenase (B) and cyclodehydratase (C and D). The ribosomal synthesis of the precursor peptide is the first step in the biosynthesis. After the generation of the enzyme-substrate complex, ATP-dependent cyclodehydration takes place, developing into an azoline heterocycle. The most characteristic feature in a LAP is the genetically and biochemically conserved installation of azol(in)e rings on non-macrocyclized natural products), however, other post-translational modifications can be found, such as acetylation, methylation, and dehydration [50]. Known LAP include streptolysin S (*Streptococcus pyogenes*), microcin B17 (*Escherichia coli*), plantazolicin (*Bacillus velezensis)*, goadsporin (*Streptomyces* sp. TP- A0584) and clostridiolysin S (*Clostridium botulinum*), owing to the difficulty inherent to the structure elucidation of heterocycles many LAPs remain unidentified [53, 54].

It remains difficult to predict the exact compound products from genome sequence data only. The functionality of the novel BGCs found in our *Paenibacillus* sp. PL91 strain remains elusive, and further studies will be necessary, including purification of the compound, High Performance Liquid Chromatography (HPLC) mass spectrometry (MS/MS) and Nuclear Magnetic Resonance (NMR) spectroscopy, as well as antimicrobial target specificity of each individual compound. These studies have already been initiated.

## Conclusions

Biological control represents an alternative safe method to counter the extensive use of chemical pesticides which has caused severe environmental and health problems.

In this study, we identified three novel potential biocontrol strains, out of 69 strains screened, that can antagonize phytopathogens and human pathogens, showing potential to be used as biocontrol agents and promote plant growth as well. Further mining into the genomes of the potential biocontrol strains the *in-silico* prediction revealed a great number of BGCs, including both known and potential novel ones. We found that BGCs in *Bacillus* species frequently encode conserved known compounds. The *Paenibacillus sp.* PL91 strain showed the largest number of possible novel BCGs; in our study we found one intact NRPs and two promising bacteriocins. Continued efforts will be directed to purify and characterize these interesting secondary metabolites as well as their determining their contribution to developing biocontrol agents.

## Methods

### Collection of plant material and bacterial isolation

Plant material used in this study came from a small farm in the town of Roden, the Netherlands. Briefly, 2 g of leaves from tomato and lettuce were macerated to a homogenous liquid state using 5ml of 10 mM MgSO4 buffer. The suspension was diluted 10^3^–10^6^ times with 10 mM MgSO4 buffer, and each dilution was exposed to heat treatment of 80 °C for 15 min and subsequently spread on LB agar plates and incubated at 28 °C for the next 72 h. Colony growth was monitored and each colony was isolated and cultured on a separate plate. Stocks were created by using glycerol at 80% solutions and stored at −80 °C [34].

### Phylogenetic analysis

The genome sequences of the selected strains used in our study were determined as described previously [34]. Genome-scale comparison of the bacterial strains and other relevant strains were conducted GTDB-Tk v1.5.0 [36]. A multiple sequence alignment of the 118 identified markers from 22 genomes was generated. It was employed to build a maximum likelihoodphylogenetic tree by using IQ-TREE multicore version 1.6.12 [55]. Substitution models LG+F+R4 was selected by ModelFinder [56]. iTOL web-based tool [36] was used for tree visualization, using the previous alignment.

## Antimicrobial activity assays

### Fungal inhibition assay

Each fungus was inoculated in a PDA agar plate and incubated at 28 °C for specific time according to the fungus growth rate. *Rhizoctonia solani*, *Phytium ultimum*, *Aspergillus Niger* and *Fusarium culmorum* 3 days of incubation, and Botrytis cinerea, *Alternaria Solani* and *Fusarium oxysporum* 5 days of incubation. Briefly, an agar plug (5 mm diameter) with fungal hyphae was inoculated into a PDA plate at the center, and 5 µl of 1 × 10^8^ cells/ml overnight culture of each isolated strain of the collection isolates, were subsequently spotted 2 cm away from the plug in 4 corners symmetrically. Plates were sealed with parafilm and incubated at 28 °C for each fungus specific growth time, and inhibition was documented.

### *Verticillum dahliae* and *Septoria licopersici* fungal inhibition assay

Slow growing fungi *Verticullium dahliae* and *Septoria licopersici* were incubated for one month at 28 °C. Plugs were cutted from 3 whole plates with each different fungus, and then vortexed in 10 ml of sterile water to collect the spores, mixed with 15 ml PDA agar, and the solution poured into plates. After drying 5 µl of 1 × 10^8^ cells/ml overnight culture of *SPL51, STRP31 andPL91* strain was spotted at the center, followed by two to eight days incubation at 28 °C, and inhibition was registered.

### Bacterial inhibition assay

To test antibacterial activity, overnight cultures of bacterial pathogens were grown until OD_600_= 1.0; then mixed with LB agar media, at a final concentration of 1 × 10^6^ cells/ml. The mixed media was poured into petri dishes to get pathogen- fusion agar plates. Subsequently 7 µl of (1 × 10^8^ cells /mL) culture of each isolated strain was inoculated at the center of the plate, followed by incubation between 24 and 48 h. The presence or absence of a halo was monitored, and the inhibition zone was determined in (mm).

### Plant growth promoting assay by volatile compounds (VOCs) in vivo

Tomato seeds were sterilized by submerging them in 2% sodium hypochlorite for 15 min and then washed with sterile water 5 times to remove completely sodium hypochlorite. Seeds were germinated on large Petri dishes with Murashigue Skoog medium (Duchefa Biochemie) and incubated in a climate chamber with the next conditions: Light-time (16 h) at 24+/- 2 Celsius degrees, 4000-6000 lx (or photon flux density of 200 umol m-2 S-1); Dark time (8 h) at 21+/-2 Celsius degrees; humility 75+/-5%. Once seeds germinated after 3 or 6 days (lettuce and tomato respectively), seedlings were transferred to a new MS Petri dish and 10 µl of overnight cultures with OD_600_ 1.0 were inoculated onto a separate small petridish inside the MS plate creating a separate compartment in order to evaluate plant growth promotion by volatile compounds. Plates were sealed with parafilm and after co-culture of 7 and 10 days (lettuce and tomato respectively) in the same conditions mentioned before, fresh weight and dry weight of the plants was measured. All methods were performed in accordance with the relevant guidelines/regulations/legislation. A one-way ANOVA analysis using a post-hoc Tukey HSD test was conducted (*P* < 0.05) to evaluate the significance.

### Genome mining for BGCs

The genome mining of biosynthetic gene clusters of antimicrobial compounds was conducted with antiSMASH 5.0 [57] and BAGEL4 [58]. Before applying to the pipelines, the draft genome of each strain which was originally assembled with Spades [35] was re-assembled into a pseudomolecule using closely related strains as a reference using Medusa web server. When a draft genome is created, individual reads of DNA are second assembled into contigs, which, have gaps between them. Medusa web server bridge the gaps between these contigs to create a scaffold, is a draft genome scaffolder that uses multiple reference genomes in a graph-based approach to determine the correct order and orientation of the contigs [59] (http://combo.dbe.unifi.it/medusa). The genes predicted from both pipelines were further confirmed with protein BLAST. BGCs that showed equal or less than 80% protein identity to the reported ones were regarded as novel.

## Supplementary Information

**Additional file 1.**

## Data Availability

The whole genome data are available at DDBJ/EMBL/GenBank under the bio project accession PRJNA623850 ( https://www.ncbi.nlm.nih.gov/bioproject/623850 ).

## Abbreviations

BCAs: Biocontrol agents
BGCs: Biosynthetic gene clusters
NRPs: Nonribosomal peptides
NRPSs: Nonribosomal peptide synthetases
PKs: Polyketides
PKSs: Polyketide synthetases
RiPPs: Ribosomally produced and posttranslationally modified peptides
VOCs: Volatile organic compounds
A: Adenylation
ACP: Acyl-carrier protein
AT: Acyltransferase
Atd: Trans-acyltransferase docking
C: Condensation
CAL: Co-enzyme A ligase domain
DH: Dehydratase
E: Epimerization
ISR: Induced systemic resistance
KR: Keto-reductase
KS: Keto-synthase
PCP: Peptidyl carrier protein
PGPR: Plant growth-promoting rhizobacteria
TE: Thioesterase
