# Supplementary material for: Biocontrol properties from phyllospheric bacteria isolated from Solanum lycopersicum and Lactuca sativa and genome mining of antimicrobial gene clusters
Source: BMC Genomics. 2022 Feb 21;23:152. doi: 10.1186/s12864-022-08392-0 (PMC8862347; doi:10.1186/s12864-022-08392-0)
Supplement: Supplementary file 1 — Additional file 1. [file 12864_2022_8392_MOESM1_ESM.pdf]

Supplementary data

Table S1. Strains isolated from tomato and lettuce phyllosphere

	COUNTRY	CITY	CROP	SECTION
PL1	Netherlands	Groningen	Lettuce	Leaf
PL2	Netherlands	Groningen	Lettuce	Leaf
PL3	Netherlands	Groningen	Lettuce	Leaf
PL4	Netherlands	Groningen	Lettuce	Leaf
PL5	Netherlands	Groningen	Lettuce	Leaf
PL6	Netherlands	Groningen	Lettuce	Leaf
PL7	Netherlands	Groningen	Lettuce	Leaf
PL8	Netherlands	Groningen	Lettuce	Leaf
PL9	Netherlands	Groningen	Lettuce	Leaf
PL10	Netherlands	Groningen	Lettuce	Leaf
PL11	Netherlands	Groningen	Lettuce	Leaf
PL12	Netherlands	Groningen	Lettuce	Leaf
PL13	Netherlands	Groningen	Lettuce	Leaf
PL14	Netherlands	Groningen	Lettuce	Leaf
PL15	Netherlands	Groningen	Lettuce	Leaf
PL16	Netherlands	Groningen	Lettuce	Leaf
PL17	Netherlands	Groningen	Lettuce	Leaf
PL18	Netherlands	Groningen	Lettuce	Leaf
PL19	Netherlands	Groningen	Lettuce	Leaf
PL20	Netherlands	Groningen	Lettuce	Leaf
SPL1	Netherlands	Groningen	Lettuce	Leaf
SPL2	Netherlands	Groningen	Lettuce	Leaf
SPL3	Netherlands	Groningen	Lettuce	Leaf
SPL4	Netherlands	Groningen	Lettuce	Leaf
SPL5	Netherlands	Groningen	Lettuce	Leaf
SPL6	Netherlands	Groningen	Lettuce	Leaf
SPL7	Netherlands	Groningen	Lettuce	Leaf
SPL8	Netherlands	Groningen	Lettuce	Leaf
SPL9	Netherlands	Groningen	Lettuce	Leaf
SPL10	Netherlands	Groningen	Lettuce	Leaf
SPL11	Netherlands	Groningen	Lettuce	Leaf
SPL12	Netherlands	Groningen	Lettuce	Leaf
L1	Netherlands	Groningen	Lettuce	Leaf
L2	Netherlands	Groningen	Lettuce	Leaf

L3	Netherlands	Groningen	Lettuce	Leaf
L4	Netherlands	Groningen	Lettuce	Leaf
L5	Netherlands	Groningen	Lettuce	Leaf
L6	Netherlands	Groningen	Lettuce	Leaf
STP1	Netherlands	Groningen	Tomato	Leaf
STP2	Netherlands	Groningen	Tomato	Leaf
STP3	Netherlands	Groningen	Tomato	Leaf
STP4	Netherlands	Groningen	Tomato	Leaf
STP5	Netherlands	Groningen	Tomato	Leaf
STP6	Netherlands	Groningen	Tomato	Leaf
STP7	Netherlands	Groningen	Tomato	Leaf
STP8	Netherlands	Groningen	Tomato	Leaf
STP9	Netherlands	Groningen	Tomato	Leaf
STP10	Netherlands	Groningen	Tomato	Leaf
STP11	Netherlands	Groningen	Tomato	Leaf
STP12	Netherlands	Groningen	Tomato	Leaf
STP13	Netherlands	Groningen	Tomato	Leaf
STP14	Netherlands	Groningen	Tomato	Leaf
STP15	Netherlands	Groningen	Tomato	Leaf
STP16	Netherlands	Groningen	Tomato	Leaf
STP17	Netherlands	Groningen	Tomato	Leaf
STP18	Netherlands	Groningen	Tomato	Leaf
STP19	Netherlands	Groningen	Tomato	Leaf
STP20	Netherlands	Groningen	Tomato	Leaf
STP21	Netherlands	Groningen	Tomato	Leaf
STP22	Netherlands	Groningen	Tomato	Leaf
STP23	Netherlands	Groningen	Tomato	Leaf
STP24	Netherlands	Groningen	Tomato	Leaf
STP25	Netherlands	Groningen	Tomato	Leaf
STP26	Netherlands	Groningen	Tomato	Leaf
STP27	Netherlands	Groningen	Tomato	Leaf
STP28	Netherlands	Groningen	Tomato	Leaf
STP29	Netherlands	Groningen	Tomato	Leaf
STP30	Netherlands	Groningen	Tomato	Leaf
STP31	Netherlands	Groningen	Tomato	Leaf

Table S2. Known antimicrobials BGCs found in the genomes of the selected strains.

Strain	Size (bp)	Type	Antimicrobial compound
B. velezensis SPL51	42962	NRPS	Anabaenopeptin
	41418	NRPS	Bacilysin
	50509	NRPS	Bacillibactin
	105756	PKS	Difficidin
	134055	NRPS	Fengycin
	108799	PKS	Bacillaene
	88230	PKS	Macrolactin H
	23177	Bacteriocin	Plantazolicin
	64641	NRPS	Surfactin
B. subtilis STRP31	24457	Bacteriocin	Subtilomycin
	63029	NRPS	Surfactin
	114771	PKS	Bacillaene
	80032	NRPS	Fengycin
	49741	NRPS	Bacillibactin
	21611	Bacteriocin	Subtilosin A
	41418	NRPS	Bacilysin

Table S3. Accession number of selected genomes for phylogenetic analysis

Bacillus subtilis subsp. subtilis str. 168	GCA_000009045.1
Brevibacillus brevis NBRC 100599	GCA_000010165.1
Bacillus licheniformis ATCC 14580	GCA_000011645.1
Bacillus velezensis FZB42	GCA_000015785.2
Paenibacillus JDR-2	GCA_000023585.1
Bacillus amyloliquefaciens DSM7	GCA_000196735.1
Bacillus thuringiensis YBT-1518	GCA_000497525.2
Bacillus velezensis SQR9	GCA_000685725.1
Paenibacillus polymyxa CF05	GCA_000785455.1
Bacillus megaterium NBRC 15308	GCA_000832985.1
Bacillus mycoides ATCC 6462	GCA_000832605.1
Paenibacillus beijingensis DSM 24997	GCA_000961095.1
Bacillus pumilus SH-B9	GCA_001578205.1
Paenibacillus ferrarius CY1	GCA_002027705.1
Bacillus subtilis NCD-2	GCA_002556525.1
Bacillus cereus ATCC 14579	GCA_006094295.1
Paenibacillus luteus	GCA_006542765.1
Bacillus velezensis SPL51	GCA_012935315.1
Paenibacillus sp. PL91	GCA_012935325.2
Bacillus subtilis STRP31	GCA_012935335.1
Bacillus amyloliquefaciens WFO2	GCA_013122255.1
Paenibacillus endophyticus CECT 8234	GCA_014192395.1
